# The effect of a trimetallic nanocomposite in the solar absorber layer of organic solar cells†

**DOI:** 10.1039/c8ra08725c

**Published:** 2019-02-19

**Authors:** Xolani G. Mbuyise, Elhadi A. A. Arbab, Genene Tessema Mola

**Affiliations:** School of Chemistry & Physics, University of KwaZulu-Natal Pietermaritzburg Campus, Private Bag X01 Scottsville 3209 South Africa mola@ukzn.ac.za; Department of Physics, Faculty of Science and Technology, Omdurman Islamic University Omdurman P.O. Box 382 Sudan

## Abstract

Bulk heterojunction (BHJ) organic solar cells were fabricated using a trimetallic nanocomposite (Ag : Zn : Ni) in the photoactive layer. The incorporation of the nanocomposite was limited to the concentrations of 4% and 6% by volume into poly(3-hexylthiophene) (P3HT) and 6-6-phenyl-C_61_-butyric acid methyl ester (PCBM) blend solar absorber. The newly fabricated devices were investigated in terms of the optical, electrical and morphological properties of the photoactive medium. The power conversion efficiencies (PCE) of the solar cells were found to be increased by 57% and 84% due to improved harvesting of solar radiation due to the occurrence of localized surface plasmon resonance (LSPR) effects of the metal nanocomposite. Silver : zinc : nickel (Ag : Zn : Ni) tri-metallic nanocomposites were synthesized using a chemical reduction method from silver, zinc and nickel nitrates. The nanocomposites were characterized in terms of morphology, elemental composition and crystallinity which are extensively discussed in the manuscript.

## Abstract 2

Bulk heterojunction (BHJ) organic solar cells were fabricated using a trimetallic nanocomposite (Ag : Zn : Ni) in the photoactive layer.

## Graphical abstract

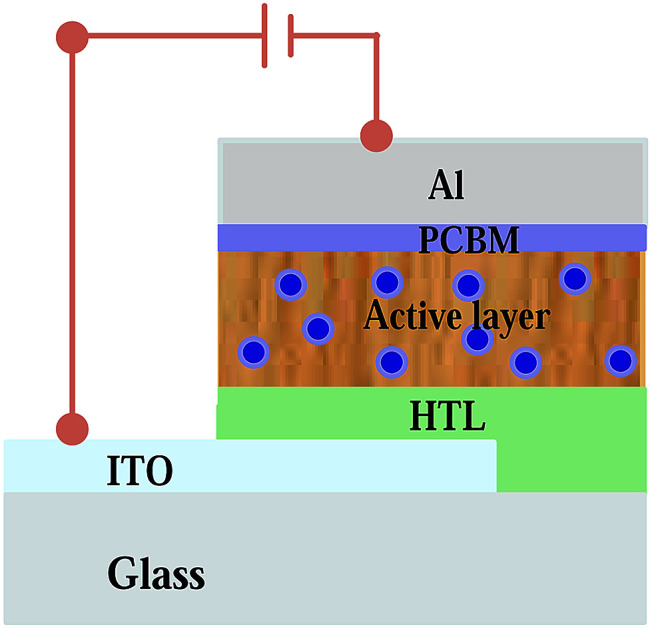

## 1 Introduction

The global energy demand for more and sustainable energy sources has increasingly led researchers to shift their attention towards clean and renewable energy sources.^1–3^ Solar energy is one of the most abundant energy sources that can be converted into electrical energy by means of solar cells. In an effort to maximize the harvesting of solar energy, a number of solar cell technologies have been developed since the 1950s with the view to alleviate the challenges that our society is facing today, in terms of the need for more energy and environmental pollution. Organic photovoltaic (OPV) cells are one of the solar cell technologies that have recently emerged and attracted remarkable attention in energy research. OPVs offer a number of advantages such as low cost device fabrication, light weight and flexibility compared to the conventional silicon based inorganic solar cells.^4–7^ Organic photovoltaic cells have been fabricated in a number of designs to be able to improve the power conversion efficiencies (PCE) and environment stabilities of the devices. The most successful OPV architecture is the bulk heterojunction (BHJ) design, in which the active layer is composed of donor and acceptor organic molecules blend, to fabricate an efficient thin film solar cell.^8,9^ In a BHJ, the donor and acceptor molecules in the photoactive medium form phase separated domains, which serve as an effective dissociation centres for photon generated excitons, due to an increased number of nanoscale donor/acceptor interfaces. The distribution of interfacial domains, with sizes comparable to the exciton diffusion length in the polymer medium, would create better chances for exciton dissociation into free charge carriers.^10^ Research into the incorporation of metal and/or semiconducting nanocomposites in organic photovoltaic cells has found several advantages because of their positive contributions in harvesting solar radiation. This includes but not limited to local surface plasmon resonance (LSPR) effects and assistance in the charge transport processes in the medium.^10–19^

Metals and semiconductor nanocomposites have been investigated extensively in recent years for their potential applications in photonic devices. The excitation of the localized surface plasmon resonance, light trapping and the ability to act as an electron cascade of metal nanocomposites in photoactive media of OPV are the most attractive features of materials to serve as components of solar absorber. A metal nanocomposite in a polymer matrix exhibited strong local electromagnetic (EM) fields that assisted in exciton dissociation and charge mobility which eventually increased the overall device performance. Several investigations have clearly demonstrated the effects of uni- and bi-metallic nanocomposites on the optical absorption, exciton dissociation and charge transport processes in thin film organic solar cells (TFOSCs).^20–22^ However, there are no reports to date, to the knowledge of the authors, about the use of tri-metallic nanocomposites in the preparation of photonic devices. Metal nanocomposites were incorporated and tested in the various functional layers of TFOSCs to achieve the optimum conditions for high device performance.^8,23,24^ This article reports on the synthetic routes for tri-metallic nanocomposites (Ag : Zn : Ni) and its application in the solar absorber of thin film organic solar cells. The nanocomposite containing silver, zinc and nickel in the photoactive layer does not only increase the conductivity of the medium but also generates free charge carriers upon interaction with the incident radiation. Employing the Ag : Zn : Ni nanocomposite as a dopant in a P3HT:PCBM blend photoactive layer showed an improved short-circuit current (*J*_sc_) and fill factor (FF). UV-Vis and XRD measurements were also conducted to understand the optical and structural properties of the Ag : Zn : Ni nanocomposite.

## 2 Materials and methods

### 2.1. Synthesis of trimetallic nanocomposites

Ag : Zn : Ni tri-NCs were synthesized using a wet chemical processing method. A solution composed of 40 mM of silver nitrate, 20 mM of zinc nitrate and 20 mM of nickel nitrate was prepared using deionized water. 0.5 M sodium borohydride was added to serve as the reducing agent. The solutions were mixed together in a beaker starting with the silver nitrate solution, followed by a dropwise addition of the remaining solution under vigorous stirring. The mixture was stirred continuously for 3 to 4 hours at a temperature of 40 °C. The resultant suspension was then filtered and rinsed several times with deionized water to wash out the sodium nitrate and to ensure pure metallic nanoparticles. The Ag : Zn : Ni tri-NCs were then characterized using a UV-Vis absorption spectrometer (T80-PG-Instrument limited), a high-resolution transmission electron microscope (HR-TEM: JEOL JEM 2100), a scanning electron microscope (SEM: JEOL JSM 6100) and a Bruker D8 Advance X-ray powder diffractometer with high-intensity Cu K radiation (=0.15406 nm).

### 2.2. Device preparation

**Fig. 1:**
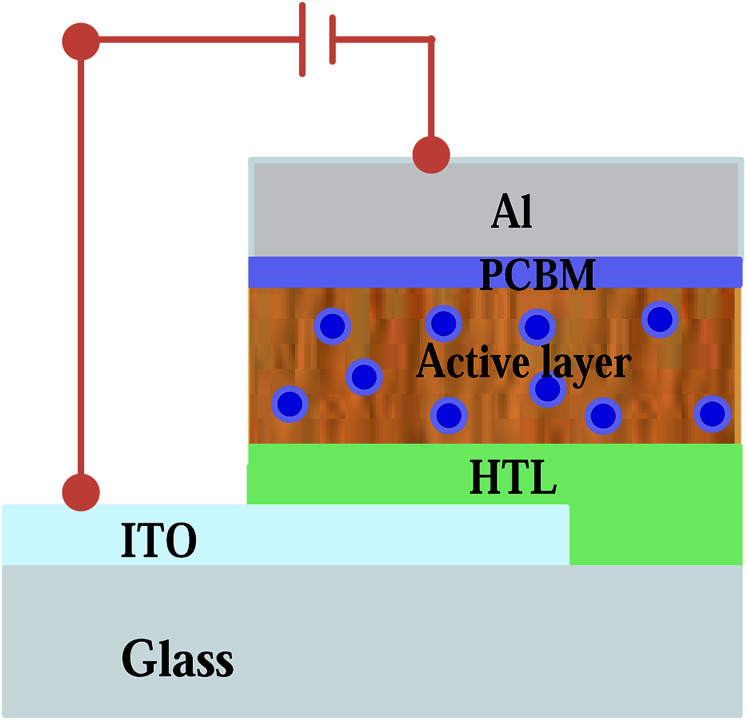
Schematic diagram of the thin film organic solar cell with the Ag : Zn : Ni nanocomposite incorporated into the photoactive medium.

Chemicals and ITO coated glass substrate (sheet resistance of 15 Ω sq^−1^) were purchased from Ossila Ltd. and used as delivered. The fabrication of thin film organic solar cell (TFOSCs) begins by partially etching the ITO substrates with acid solution containing HCl : H_2_O : HNO_3_ at the concentration ratio 48% : 48% : 4% by volume. Then, the substrates were thoroughly cleaned in deionized water, acetone and isopropanol using an ultrasonic bath for 10 minutes each, respectively. They were then dried in an oven at 150 °C for 20 min before spin coating the hole transport layer (HTL). The solution of the hole transport layer PEDOT:PSS was spin coated on the substrates at 3500 rpm for 60 seconds. The HTL coated substrates were annealed again in an oven at 150 °C for 30 min. The photoactive layer of the solar cells was prepared in chloroform solvent containing poly(3-hexylthiophene) (P3HT) and 6-6-phenyl-C_61_-butyric acid methyl ester (PCBM) blend at a 1 : 1 ratio by weight. The concentration of the pristine solution was 20 mg ml^−1^ and it was stirred for 3 h at 40 °C to enhance the miscibility of the molecules. The other two solutions were prepared with the addition of 4 vol% and 6 vol% of the Ag : Zn : Ni tri-NC to the reference P3HT:PCBM solution. The solutions were stirred on a hot plate at an average temperature of 45 °C for 5–6 hours for better miscibility of the molecules in the active layer blend. The active layers were then spin coated on top of the HTL at a rate of 1200 rpm for 40 seconds and dried in a furnace at 100 °C for 5 min under a nitrogen atmosphere. The samples were then loaded into a vacuum chamber (Edward Auto 306 deposition unit) at a base pressure of 10^−6^ mbar. Finally, a thin buffer layer of lithium fluoride (LiF) used as an electron transport layer (ETL) and an aluminium (Al) electrode were deposited on top of the active layer with thicknesses of 0.4 nm and 60 nm, respectively. TFOSC devices was fabricated based on the standard device structure ITO/PEDOT:PSS/(P3HT:PCBM doped with Ag : Zn : Ni)/LiF/Al in ambient laboratory conditions (see Fig. 1). The electrical characterization of the devices was carried out using a computer interfaced Keithley HP2400 source-meter and a solar simulator (model SS50AAA) operating at AM1.5 and an integrated power intensity of 100 mW cm^−2^. The resulting diodes had an effective area of 4 mm^2^. The charge transport properties and recombination dynamics were analysed using space charge limited current taken from the *J*–*V* data under dark conditions. The thin film absorption characteristics of the devices were studied using UV-Vis absorption spectra obtained with an absorption meter (T80-PG-Instrument limited).

## 3 Results and discussion

### 3.1. Characterization of the metal nanocomposites

#### 3.1.1. HRSEM & HRTEM

**Fig. 2:**
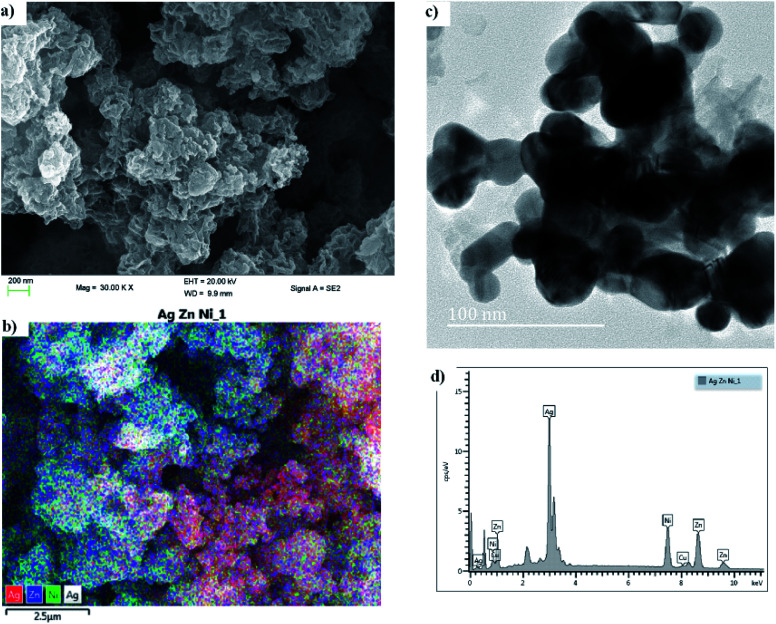
(a and b) HRSEM, and (c) HRTEM images, and (d) an energy dispersive X-ray (EDX) spectrum of Ag : Zn : Ni.

High resolution scanning and electron microscopy (HRSEM and HRTEM) was used to study the particle size and crystallinity of the synthesized Ag : Zn : Ni tri-NC. The HRTEM image given in Fig. 2b was recorded for the metal nanoparticle powder and shows the crystalline structure of the Ag : Zn : Ni tri-NC, as is also evident from the diffraction pattern obtained therefrom. The metal nanoparticles formed in various sizes and forms as indicated in the figure. The particle sizes ranged from 10 nm to 45 nm which was confirmed by X-ray diffraction experiments. The SEM images given in Fig. 2a and 2c clearly show the flower-like structures in the powder form. The energy dispersive X-ray (EDX) spectrum provided in Fig. 2d shows various characteristics peaks associated with the presence of silver, zinc and nickel in the ratio of 2 : 1 : 1, respectively. Thus, the high silver content in the Ag : Zn : Ni nanocomposite enhances the optical properties of the solar absorber films due to the LSPR effect. While the zinc and nickel present serve as light scattering centers that increases absorption in the near infrared region as depicted in Fig. 4. The elemental mapping taken from the SEM image (see Fig. 2b) indicated that there is an even distribution of the elements with a dominant presence of silver. Generally, the conductivity of the polymer medium improves significantly in the presence of the metal nanocomposite in addition to the formation of a strong electromagnetic field at the site of the metal particles. The influence of all of these factors is evident in the overall performance of the fabricated device.

#### 3.1.2. XRD

**Fig. 3:**
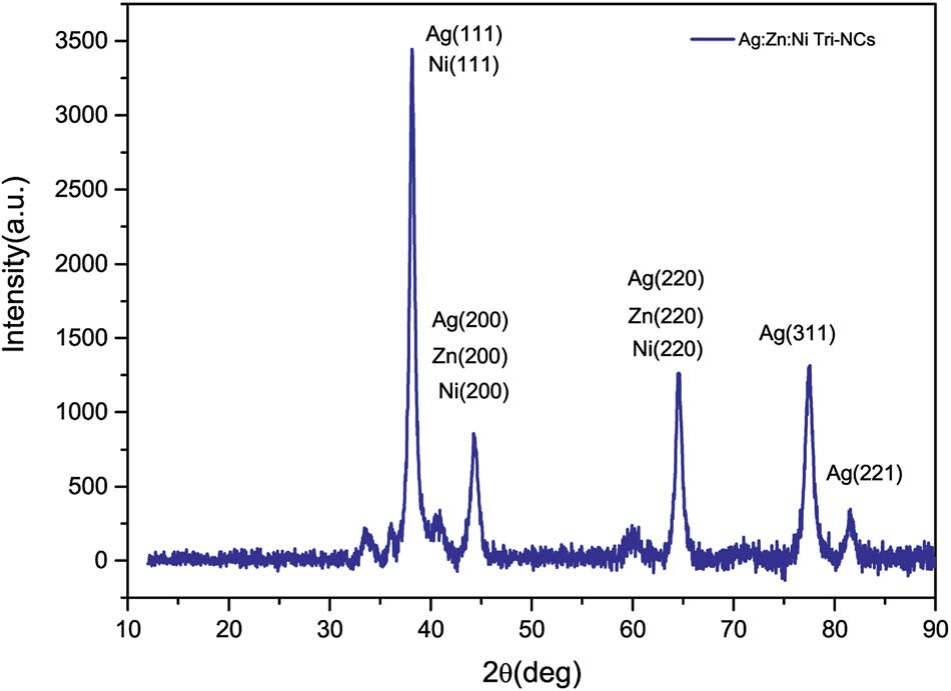
An XRD pattern of the Ag : Zn : Ni nanocomposite powder.

The crystal phase and composition of the Ag : Zn : Ni nanocomposites were characterized by an X-ray diffraction (XRD) method. Fig. 3 shows the XRD pattern of the Ag : Zn : Ni NC powder in the range of 30–90° in steps of 0.025 at a scanning speed of 20° min^−1^. A number of Bragg reflections with 2*θ* values of 38.110°, 44.280°, 64.470°, 77.430° and 81.550° are observed corresponding to the (111), (200), (220), (311), and (222) planes of Ag nanoparticles (NPs). Therefore, the observed characteristic peaks corresponds to the crystal planes (200) and (220) are due to Zn and Ni. The average particle size of the Ag : Zn : Ni tri-NC was evaluated from the width of the reflection according to Debye–Scherrer equation:^14^1
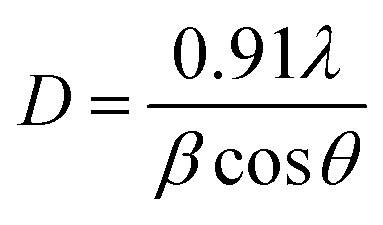
where *β* is the full width at half maximum (FWHM) of the peak in radius, *θ* is the angle of diffraction and *λ* is the wavelength of the X-ray. Using a Rich Seifert diffractometer with CuK_α_ (*λ* = 1.5418 nm), therefore, the crystalline size determined from this analysis was found to be 5.40 nm from the width of the dominant peak at the (111) crystal plane which indicated that the synthesized powder contained mixed crystallographic phases. A. Revina *et al.*^12^ have suggested that the particles having a size of less than 5 nm possess a cubic zinc blend crystal structure while particles above 5 nm in size have a mixture of both cubic and hexagonal phases. Thus, the particle size is one of the crucial parameters used to determine the crystallographic phase. All diffraction peaks were indexed according to face-centred cubic (fcc) and hexagonal phase structures. The two phases require similar energy for the transition from one phase to another. The nanoparticles appeared to be crystalline, as indicated in the diffraction patterns provided in (Fig. 2b and 3).^16,17^ It is to be noted here that we have synthesized a pure Ag : Zn : Ni nanocomposite with relative ease and low cost using a wet chemical processing method.

### 3.2. Device characterization

#### 3.2.1. UV-Vis measurements

**Fig. 4:**
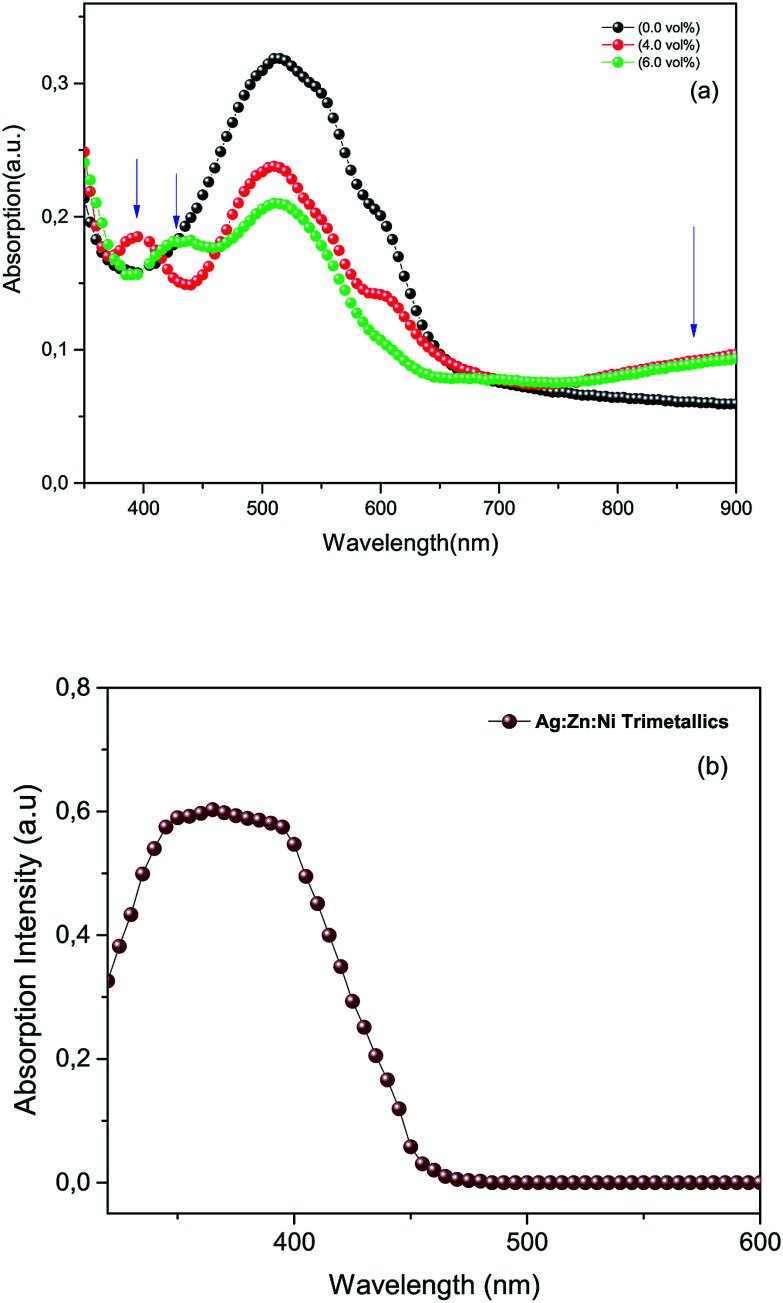
(a) UV-Vis spectra of a photoactive film reference and photoactive films doped with tri-metallic nanoparticles. The blue arrows in the top panel indicate the positions of LSPR absorption of the metal nanoparticles. (b) A UV-Vis spectrum of the tri-metallic (Ag : Zn : Ni) powder in a deionized water suspension.

Normalized UV-Vis absorbance spectra provided in Fig. 4a were recorded from photoactive films of P3HT:PCBM (reference) and P3HT:PCBM doped with the Ag : Zn : Ni nanocomposite at the concentrations of 0%, 4 vol% and 6 vol% dispersed in a chloroform based solution, respectively. The lower panel (Fig. 4b) was recorded from the Ag : Zn : Ni nanocomposite powder in a deionized water suspension. The pristine film (top panel) shows a typical absorption pattern for the P3HT:PCBM blend film ranging between 400–670 nm and has a peak maximum centred around 512 nm. Whereas those for the photoactive media containing the metal particles appeared to have a slightly blue shifted absorbance peak for the P3HT:PCBM blend (by ∼25 nm) due to the interactions of the metals with the polymer molecules.^24–27^ In addition, the doped films showed an additional absorbance at the short wavelength visible region and infrared regions (see arrows in Fig. 4a). The absorption spectra of the doped films contain two peaks in the short wavelength range centred around 390 nm and 430 nm, respectively, in addition to the peak in the infrared region. These absorption peaks are due to the LSPR effect due to the presence of metal nanoparticles in the medium (see Fig. 4b). It is to be noted here that the spectrum in Fig. 4b does not show tri-metallic resonance absorption in the infrared region in water but evidently show resonance in the polymer matrix. This could be attributed to the differences in the dielectric constants of the media.

The absorbance of the P3HT:PCBM blend generally decreased over the range from 420 to 650 nm because of the presence of the metal nanoparticles. This is due to the fact that the incorporation of the metal nanoparticles in the polymer matrix will certainly reduce the amount of interactions of P3HT:PCBM with incident photons, at least within the cross-sectional area of the spectrometer beam. This in turn will decrease the amount of absorbed photons by the P3HT:PCBM within the indicated range since the resonance absorbance of the metal nanoparticles is outside of the indicated range (*i.e.* UV and infrared). However, the attenuation of the absorbance of P3HT:PCBM (420 to 650 nm) is an indication of the good miscibility of the nanoparticles in the polymer matrix. The particles used in this experiment were not uniform in size or shape. The metal nanoparticles were synthesized by wet processing that produced particles of different sizes and shapes. As a result, the LSPR absorbance peaks could change from short wavelength visible to infrared regions depending of the nature of the particles.^27^ The enhanced absorption in the near infrared region might also be caused by the inelastic scattering of the electromagnetic radiation (EM) inside the photoactive medium that assisted the increased optical path length in the polymer matrix.^20–24,28–31^ According to the Doppler shift effect, the red shifted absorption peak of the doped device confirms the scattering effect of the synthesized silver-based nanoparticles incorporated in the photoactive medium.

### 3.3. Electrical properties of the fabricated organic solar cells

**Fig. 5:**
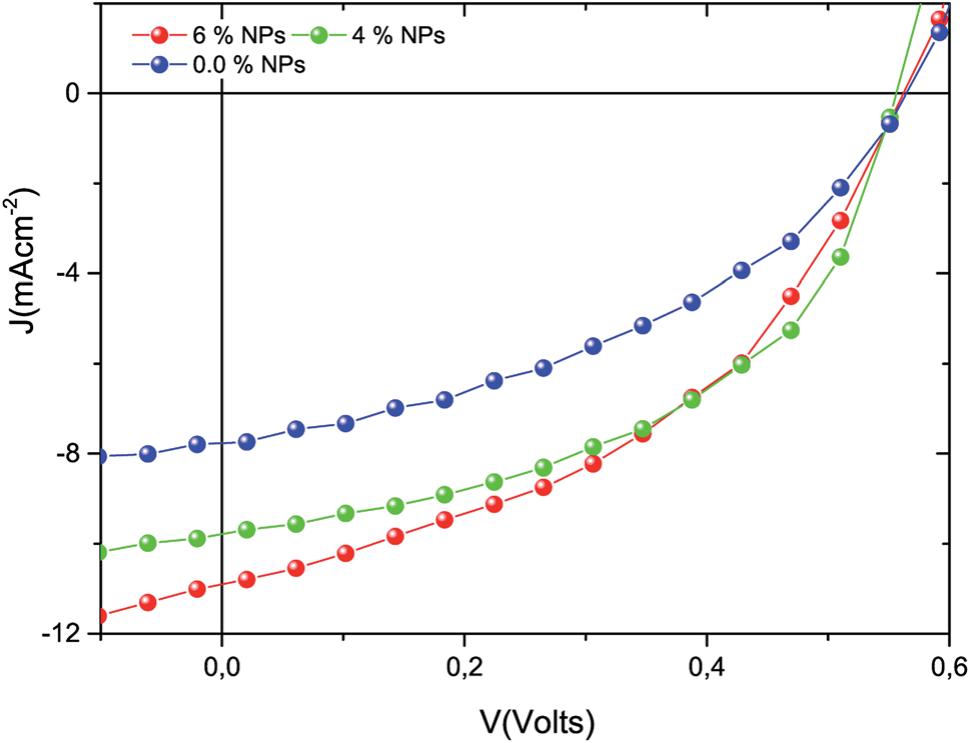
*J*–*V* characteristics of the devices produced as reference and metal nanocomposite doped solar cells.

Organic solar cells with a photoactive medium comprising P3HT:PCBM (reference) and P3HT:PCBM doped with the Ag : Zn : Ni nanocomposite were fabricated in this study. The current–voltage (*J*–*V*) characteristics recorded for the newly fabricated solar cells are given in Fig. 5. According to the *J*–*V* curves, the photocurrent measured from the solar cells with metal nanoparticles exhibited a higher magnitude than that of the pristine film solar cell. All of the solar cell parameters, derived from the measured electrical properties, are presented in Table 1, and show significant enhancement in the short circuit current density (*J*_sc_), the fill factor (FF), and the PCE for the metal nanoparticle doped devices compared to the reference solar cell.

**Table 1:** The *J*–*V* parameters of the OPV cells for the reference and the doped photoactive medium

P3HT:PCBM (Ag : Zn : Ni (vol%))	*V*_oc_ (volts)	*J*_sc_ (mA cm^−2^)	FF (%)	PCE (%)
0.0%	0.58	7.83	40.08	1.81
4.0%	0.57	10.56	47.50	2.84
6.0%	0.56	12.36	47.70	3.33

The localized surface plasmon resonance and associated light scattering effect of the tri-NC lead to the improved photon-to-electron conversion efficiency. The LSPR effects not only lead to increased light absorption of the active layer materials but also benefit the charge separation and transport, resulting in increased charge carrier density, mobility and lifetime. It is evident that cooperative plasmonic enhancement by the simple combination of the different metal nanoparticles (*i.e.*, Ag, Zn and Ni NPs) was achieved. The enhanced performances of the P3HT:PCBM doped Ag : Zn : Ni tri-NC devices are due to more favourable morphologies which support the transport of electrons and holes along the PCBM and P3HT phases. The power conversion efficiencies measured from the samples doped with the metal NC grew by about 57% and 84% for 4% and 6% tri-NC loading, respectively. Thus, the incorporation of a trimetallic nanocomposite in the active layer of a TFOSC gave rise to enhanced optical absorption, exciton generation, dissociation, and transport of charges that in turn enhanced the overall device performance.^32^

### 3.4. Charge transport properties

The electron and hole mobilities are important factors for polymer media and should be high enough to ensure high carrier transport by preventing charge recombination and build-up of space charge.^33^ The charge transport properties of the solar cells were studied using the space-charge limited current (SCLC) recorded under dark conditions without the interference of a photon induced charge concentration gradient. In the absence of pin holes and high electric fields, the current density increased quadratically with the applied bias voltage (V). Thus, the SCLC current of the ln(*J*)–*V* curves were fitted with a field dependent current density equation using Mott–Gurneys law:^18,19^2
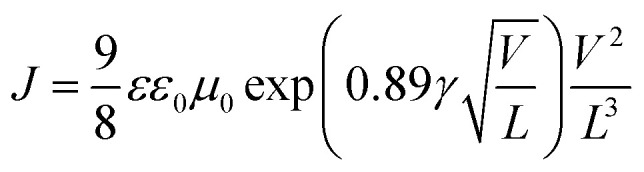
where *ε* and *ε*_0_ are the relative dielectric permittivity of the polymer medium and the free space, respectively. *L* is the thickness of the active layer, and *V* is the voltage drop across the sample. Finally, *μ*_0_ and *γ* are the zero-field mobility and the field activation factor of the medium, respectively. The results show that the zero-field mobility of the charges for the doped device was found to be one order of magnitude higher than that of the device with the pristine photoactive medium (Table 2). Thus, this is a clear indication that the presence of the tri-metallic nanocomposite enhanced the charge transport in the polymer medium. Furthermore, the addition of the Ag : Zn : Ni tri-NC in the polymer active layer may have caused the formation of additional interfacial areas that could assist in carrier transport and collection. It is to be noted here that the charge carriers have more mobility in the Ag : Zn : Ni nanocomposite than in the polymer domain, and hence, the Ag : Zn : Ni NC clusters in the medium offer alternative channels for charge percolation that improve the collection of photon generated charges.

**Table 2:** Charge transport parameters of the OPV cells fabricated with the Ag : Zn : Ni tri-NC in the photoactive medium

P3HT:PCBM (Ag : Zn : Ni (vol%))	*μ*_0_ (cm^2^ V^−1^ s^−1^)	*γ* (cm V^−1^)
0.0%	5.96 × 10^−4^	−2.2 × 10^−3^
4.0%	3.51 × 10^−3^	−1.3 × 10^−3^
6.0%	2.89 × 10^−3^	−1.8 × 10^−3^

## 4 Conclusions

A tri-metallic nanocomposite (Ag : Zn : Ni) containing Ag, Zn, and Ni was successfully synthesized using a wet processing method. The incorporation of the synthesized (Ag : Zn : Ni) NC into a P3HT:PCBM photoactive medium, of a thin film organic solar cell, improved the power conversion efficiencies of the devices by 57% and 84% for 4% and 6% volume concentrations of the suspension of the metal particles, respectively. The effects of the metal nanocomposite in the solar cells are attributed to increased light trapping within the active layer and improved charge transport processes in the BHJ films. The localised surface plasmon resonance and light scattering effects are the key players for the observed results. Finally, the Ag : Zn : Ni nanocomposite can also act as a dissociation centre and alternative transport channel to harvest photon generated currents *via* increased charge mobility that certainly improves device performance.

## Conflicts of interest

There are no conflicts of interest to declare.

## Supplementary Material
